# *catena*-Poly[[dichloridocobalt(II)]-μ-4,4′-bis­(benzimidazol-1-yl)biphen­yl]

**DOI:** 10.1107/S1600536811010671

**Published:** 2011-03-26

**Authors:** Hui Li, Qiuping Han, Chenzhong Yao, Qiaojuan Gong, Zhuangwei Wei

**Affiliations:** a Department of Applied Chemistry, Yuncheng University, Yuncheng, Shanxi 044000, People’s Republic of China

## Abstract

In the title compound, [CoCl_2_(C_26_H_18_N_4_)]_*n*_, the Co^II^ atom (site symmetry 2) is tetra­hedrally coordinated by two chloride ions and two N atoms from 4,4′-bis­(benzimidazol-1-yl)biphenyl ligands: the complete ligand is generated by crystallographic twofold symmetry. The dihedral angle between the benzene rings is 34.67 (8)° and the angle between the benene ring and the adjacent benzimidazole ring system is 43.26 (10)°. The bridging ligand links the Co^II^ atoms into chains propagating in [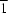
01].

## Related literature

For background to benzimidazole-based ligands in crystal engineering, see: Jin *et al.* (2006 ▶); Li *et al.* (2009 ▶); Su *et al.* (2003 ▶).
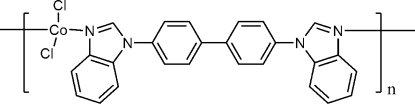


## Experimental

### 

#### Crystal data

[CoCl_2_(C_26_H_18_N_4_)]
*M* *_r_* = 516.27
Monoclinic, 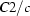

*a* = 12.878 (3) Å
*b* = 15.181 (3) Å
*c* = 11.136 (2) Å
β = 91.37 (3)°
*V* = 2176.5 (8) Å^3^
*Z* = 4
Mo *K*α radiation
μ = 1.06 mm^−1^
*T* = 293 K
0.25 × 0.20 × 0.15 mm

#### Data collection

Rigaku Mercury CCD diffractometer
Absorption correction: multi-scan (*CrystalClear*; Rigaku/MSC, 2005 ▶) *T*_min_ = 0.776, *T*_max_ = 0.853
13945 measured reflections
2696 independent reflections
2361 reflections with *I* > 2σ(*I*)
*R*_int_ = 0.053

#### Refinement

*R*[*F* ^2^ > 2σ(*F* ^2^)] = 0.053
*wR*(*F* ^2^) = 0.124
*S* = 1.12
2696 reflections
150 parameters
H-atom parameters constrained
Δρ_max_ = 0.48 e Å^−3^
Δρ_min_ = −0.71 e Å^−3^

### 

Data collection: *CrystalClear* (Rigaku/MSC, 2005 ▶); cell refinement: *CrystalClear*; data reduction: *CrystalClear*; program(s) used to solve structure: *SHELXS97* (Sheldrick, 2008 ▶); program(s) used to refine structure: *SHELXL97* (Sheldrick, 2008 ▶); molecular graphics: *SHELXTL* (Sheldrick, 2008 ▶); software used to prepare material for publication: *SHELXTL*.

## Supplementary Material

Crystal structure: contains datablocks I, global. DOI: 10.1107/S1600536811010671/hb5820sup1.cif

Structure factors: contains datablocks I. DOI: 10.1107/S1600536811010671/hb5820Isup2.hkl

Additional supplementary materials:  crystallographic information; 3D view; checkCIF report

## Figures and Tables

**Table 1:** Selected bond lengths (Å)

Co1—N1	2.022 (2)
Co1—Cl1	2.2491 (8)

**Fig. 1.**
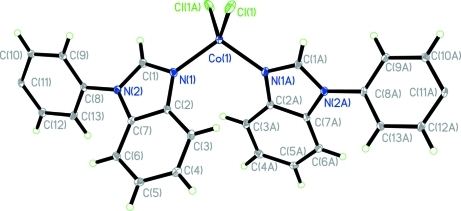
The molecular structure of (I). Displacement ellipsoids are drawn at the 30% probability level and H atoms are shown as small spheres of arbitrary radius. Atoms with suffix A are generated by (–x, y, 3/2–z).

**Fig. 2.**
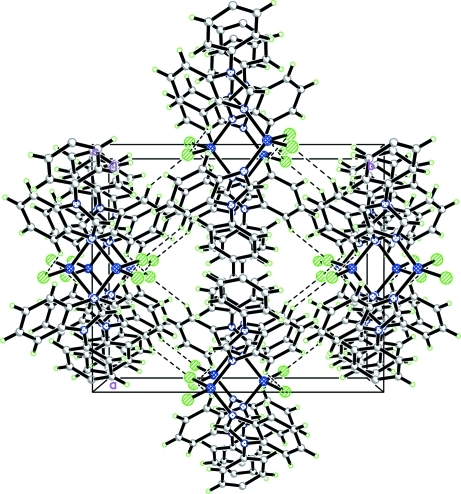
The crystal packing for (I).

## supplementary crystallographic information

### Comment

Benzimidazole has been well used in crystal engineering, and a large number of benzimidazole-containing flexible ligands have been extensively studied (Su *et al.*,2003; Jin *et al.*,2006). However, to our knowledge, the research on benzimidazole ligands bearing rigid spacers is still less developed (Li *et al.*,2009).

Single-crystal X-ray diffraction analysis reveals that the title compound (I) crystallizes in the monoclinic space group *C2/c*. The geometry of the Co^II^ ion is surrounded by two benzoiimidazole rings of distinct L ligands and two chlorine anions, which illustrates a slightly distorted tetrahedral coordination environment (Fig. 1). Notably, as shown in Fig. 2, the four-coordinated Co^II^ center is bridged by the linear ligand L to form an infinite one-dimensional architecture. The dihedral angle between the biphenyl rings is 34.67 (8)°.

### Experimental

A mixture of CH_3_OH and CHCl_3_ (1:1, 8 ml), as a buffer layer, was carefully layered over a solution of 4,4'-Bis(benzimidazol-1-yl)terphenyl (L, 0.06 mmol) in CHCl_3_ (6 ml). Then a solution of CoCl_2_ (0.06 mmol) in CH_3_OH (6 ml) was layered over the buffer layer, and the resultant reaction was left to stand at room temperature. After *ca* three weeks, blue block single crystals appeared at the boundary. Yield: ~40% (based on L).

### Refinement

C-bound H atoms were positioned geometrically and refined in the riding-model approximation, with C—H = 0.93Å and *U*_iso_(H) = 1.2Ueq.

### Crystal data

**Table:**

[CoCl_2_(C_26_H_18_N_4_)]	*F*(000) = 1052
*M**_r_* = 516.27	*D*_x_ = 1.576 Mg m^−^^3^
Monoclinic, *C*2/*c*	Mo *K*α radiation, λ = 0.71073 Å
Hall symbol: -C 2yc	Cell parameters from 3011 reflections
*a* = 12.878 (3) Å	θ = 2.1–28.3°
*b* = 15.181 (3) Å	µ = 1.06 mm^−^^1^
*c* = 11.136 (2) Å	*T* = 293 K
β = 91.37 (3)°	Block, blue
*V* = 2176.5 (8) Å^3^	0.25 × 0.20 × 0.15 mm
*Z* = 4	

### Data collection

**Table:**

Rigaku Mercury CCD diffractometer	2696 independent reflections
Radiation source: fine-focus sealed tube	2361 reflections with *I* > 2σ(*I*)
graphite	*R*_int_ = 0.053
Detector resolution: 9 pixels mm^-1^	θ_max_ = 28.3°, θ_min_ = 2.1°
ω scans	*h* = −17→17
Absorption correction: multi-scan (*CrystalClear*; Rigaku/MSC, 2005)	*k* = −20→20
*T*_min_ = 0.776, *T*_max_ = 0.853	*l* = −14→14
13945 measured reflections	

### Refinement

**Table:**

Refinement on *F*^2^	Primary atom site location: structure-invariant direct methods
Least-squares matrix: full	Secondary atom site location: difference Fourier map
*R*[*F*^2^ > 2σ(*F*^2^)] = 0.053	Hydrogen site location: inferred from neighbouring sites
*wR*(*F*^2^) = 0.124	H-atom parameters constrained
*S* = 1.12	*w* = 1/[σ^2^(*F*_o_^2^) + (0.0551*P*)^2^ + 4.8457*P*] where *P* = (*F*_o_^2^ + 2*F*_c_^2^)/3
2696 reflections	(Δ/σ)_max_ < 0.001
150 parameters	Δρ_max_ = 0.48 e Å^−^^3^
0 restraints	Δρ_min_ = −0.71 e Å^−^^3^

### Special details

**Table:**

Geometry. All e.s.d.'s (except the e.s.d. in the dihedral angle between two l.s. planes) are estimated using the full covariance matrix. The cell e.s.d.'s are taken into account individually in the estimation of e.s.d.'s in distances, angles and torsion angles; correlations between e.s.d.'s in cell parameters are only used when they are defined by crystal symmetry. An approximate (isotropic) treatment of cell e.s.d.'s is used for estimating e.s.d.'s involving l.s. planes.
Refinement. Refinement of *F*^2^ against ALL reflections. The weighted *R*-factor *wR* and goodness of fit *S* are based on *F*^2^, conventional *R*-factors *R* are based on *F*, with *F* set to zero for negative *F*^2^. The threshold expression of *F*^2^ > σ(*F*^2^) is used only for calculating *R*-factors(gt) *etc*. and is not relevant to the choice of reflections for refinement. *R*-factors based on *F*^2^ are statistically about twice as large as those based on *F*, and *R*- factors based on ALL data will be even larger.

### Fractional atomic coordinates and isotropic or equivalent isotropic displacement parameters (Å^2^)

**Table:**

	*x*	*y*	*z*	*U*_iso_*/*U*_eq_	
Co1	0.0000	0.40291 (3)	0.7500	0.01602 (16)	
Cl1	0.03587 (6)	0.32578 (5)	0.91836 (7)	0.0311 (2)	
N2	0.24698 (16)	0.52363 (14)	0.58018 (19)	0.0139 (4)	
N1	0.11781 (16)	0.48022 (14)	0.69588 (19)	0.0151 (4)	
C8	0.31898 (18)	0.52081 (17)	0.4838 (2)	0.0134 (5)	
C11	0.46103 (18)	0.51614 (17)	0.2986 (2)	0.0140 (5)	
C3	0.1150 (2)	0.61615 (17)	0.8273 (2)	0.0162 (5)	
H3	0.0601	0.5985	0.8743	0.019*	
C12	0.4046 (2)	0.59193 (17)	0.3225 (2)	0.0169 (5)	
H12	0.4150	0.6419	0.2761	0.020*	
C7	0.23231 (19)	0.59156 (16)	0.6623 (2)	0.0139 (5)	
C13	0.3332 (2)	0.59502 (17)	0.4139 (2)	0.0172 (5)	
H13	0.2955	0.6461	0.4278	0.021*	
C1	0.17714 (19)	0.45999 (17)	0.6047 (2)	0.0153 (5)	
H1	0.1715	0.4075	0.5620	0.018*	
C10	0.44322 (18)	0.44110 (17)	0.3680 (2)	0.0145 (5)	
H10	0.4781	0.3890	0.3514	0.017*	
C9	0.37368 (19)	0.44371 (17)	0.4617 (2)	0.0151 (5)	
H9	0.3638	0.3942	0.5093	0.018*	
C2	0.15099 (19)	0.56315 (17)	0.7347 (2)	0.0138 (5)	
C6	0.2823 (2)	0.67174 (17)	0.6813 (2)	0.0173 (5)	
H6	0.3365	0.6901	0.6336	0.021*	
C4	0.1643 (2)	0.69603 (18)	0.8462 (2)	0.0193 (5)	
H4	0.1423	0.7328	0.9073	0.023*	
C5	0.2473 (2)	0.72257 (17)	0.7745 (2)	0.0185 (5)	
H5	0.2795	0.7762	0.7905	0.022*	

### Atomic displacement parameters (Å^2^)

**Table:**

	*U*^11^	*U*^22^	*U*^33^	*U*^12^	*U*^13^	*U*^23^
Co1	0.0152 (3)	0.0153 (3)	0.0178 (3)	0.000	0.00656 (19)	0.000
Cl1	0.0347 (4)	0.0324 (4)	0.0267 (4)	0.0187 (3)	0.0143 (3)	0.0116 (3)
N2	0.0139 (10)	0.0151 (10)	0.0127 (10)	−0.0009 (8)	0.0033 (8)	−0.0021 (8)
N1	0.0146 (10)	0.0170 (10)	0.0138 (10)	−0.0027 (8)	0.0039 (8)	−0.0005 (8)
C8	0.0113 (10)	0.0178 (12)	0.0111 (11)	−0.0016 (9)	0.0032 (9)	0.0000 (9)
C11	0.0112 (11)	0.0184 (12)	0.0126 (12)	−0.0020 (9)	0.0041 (9)	0.0012 (9)
C3	0.0151 (11)	0.0207 (13)	0.0130 (12)	0.0016 (9)	0.0029 (10)	0.0012 (10)
C12	0.0169 (12)	0.0189 (12)	0.0152 (12)	0.0011 (9)	0.0045 (10)	0.0044 (10)
C7	0.0121 (11)	0.0163 (12)	0.0135 (12)	0.0027 (9)	0.0018 (9)	−0.0005 (9)
C13	0.0180 (12)	0.0177 (12)	0.0161 (12)	0.0046 (10)	0.0063 (10)	0.0030 (10)
C1	0.0168 (12)	0.0170 (12)	0.0124 (12)	−0.0024 (9)	0.0036 (10)	−0.0005 (9)
C10	0.0117 (11)	0.0144 (11)	0.0175 (13)	−0.0001 (9)	0.0002 (9)	−0.0015 (10)
C9	0.0158 (11)	0.0151 (12)	0.0144 (12)	−0.0029 (9)	0.0022 (9)	0.0018 (9)
C2	0.0129 (11)	0.0169 (12)	0.0116 (12)	−0.0007 (9)	0.0017 (9)	0.0006 (9)
C6	0.0153 (12)	0.0162 (12)	0.0205 (13)	−0.0013 (9)	0.0023 (10)	0.0036 (10)
C4	0.0216 (13)	0.0185 (13)	0.0177 (13)	0.0036 (10)	0.0009 (10)	−0.0023 (10)
C5	0.0214 (12)	0.0129 (11)	0.0214 (14)	0.0015 (10)	0.0011 (11)	0.0018 (10)

### Geometric parameters (Å, °)

**Table:**

Co1—N1	2.022 (2)	C3—H3	0.9300
Co1—N1^i^	2.022 (2)	C12—C13	1.388 (4)
Co1—Cl1^i^	2.2491 (8)	C12—H12	0.9300
Co1—Cl1	2.2491 (8)	C7—C6	1.391 (3)
N2—C1	1.352 (3)	C7—C2	1.404 (3)
N2—C7	1.394 (3)	C13—H13	0.9300
N2—C8	1.436 (3)	C1—H1	0.9300
N1—C1	1.321 (3)	C10—C9	1.392 (4)
N1—C2	1.395 (3)	C10—H10	0.9300
C8—C13	1.384 (3)	C9—H9	0.9300
C8—C9	1.391 (3)	C6—C5	1.378 (4)
C11—C12	1.390 (4)	C6—H6	0.9300
C11—C10	1.399 (3)	C4—C5	1.408 (4)
C11—C11^ii^	1.494 (5)	C4—H4	0.9300
C3—C4	1.383 (4)	C5—H5	0.9300
C3—C2	1.396 (4)		
			
N1—Co1—N1^i^	109.02 (13)	N2—C7—C2	105.3 (2)
N1—Co1—Cl1^i^	101.21 (7)	C8—C13—C12	118.9 (2)
N1^i^—Co1—Cl1^i^	114.22 (7)	C8—C13—H13	120.5
N1—Co1—Cl1	114.22 (7)	C12—C13—H13	120.5
N1^i^—Co1—Cl1	101.21 (7)	N1—C1—N2	112.9 (2)
Cl1^i^—Co1—Cl1	117.25 (5)	N1—C1—H1	123.6
C1—N2—C7	107.1 (2)	N2—C1—H1	123.6
C1—N2—C8	125.1 (2)	C9—C10—C11	120.5 (2)
C7—N2—C8	127.8 (2)	C9—C10—H10	119.7
C1—N1—C2	105.6 (2)	C11—C10—H10	119.7
C1—N1—Co1	123.10 (18)	C8—C9—C10	119.6 (2)
C2—N1—Co1	131.16 (17)	C8—C9—H9	120.2
C13—C8—C9	120.7 (2)	C10—C9—H9	120.2
C13—C8—N2	119.5 (2)	N1—C2—C3	130.1 (2)
C9—C8—N2	119.7 (2)	N1—C2—C7	109.1 (2)
C12—C11—C10	118.4 (2)	C3—C2—C7	120.8 (2)
C12—C11—C11^ii^	120.15 (16)	C5—C6—C7	116.5 (2)
C10—C11—C11^ii^	121.49 (16)	C5—C6—H6	121.8
C4—C3—C2	117.3 (2)	C7—C6—H6	121.8
C4—C3—H3	121.3	C3—C4—C5	121.1 (2)
C2—C3—H3	121.3	C3—C4—H4	119.5
C13—C12—C11	121.8 (2)	C5—C4—H4	119.5
C13—C12—H12	119.1	C6—C5—C4	122.2 (3)
C11—C12—H12	119.1	C6—C5—H5	118.9
C6—C7—N2	132.6 (2)	C4—C5—H5	118.9
C6—C7—C2	122.0 (2)		
			
N1^i^—Co1—N1—C1	−143.7 (2)	C8—N2—C1—N1	−177.9 (2)
Cl1^i^—Co1—N1—C1	−23.0 (2)	C12—C11—C10—C9	−2.5 (4)
Cl1—Co1—N1—C1	104.0 (2)	C11^ii^—C11—C10—C9	177.1 (3)
N1^i^—Co1—N1—C2	31.89 (19)	C13—C8—C9—C10	−0.2 (4)
Cl1^i^—Co1—N1—C2	152.6 (2)	N2—C8—C9—C10	179.8 (2)
Cl1—Co1—N1—C2	−80.5 (2)	C11—C10—C9—C8	2.2 (4)
C1—N2—C8—C13	135.7 (3)	C1—N1—C2—C3	179.4 (3)
C7—N2—C8—C13	−41.8 (4)	Co1—N1—C2—C3	3.3 (4)
C1—N2—C8—C9	−44.3 (4)	C1—N1—C2—C7	0.3 (3)
C7—N2—C8—C9	138.2 (3)	Co1—N1—C2—C7	−175.87 (17)
C10—C11—C12—C13	1.0 (4)	C4—C3—C2—N1	179.3 (2)
C11^ii^—C11—C12—C13	−178.6 (3)	C4—C3—C2—C7	−1.6 (4)
C1—N2—C7—C6	178.6 (3)	C6—C7—C2—N1	−178.9 (2)
C8—N2—C7—C6	−3.6 (4)	N2—C7—C2—N1	−0.3 (3)
C1—N2—C7—C2	0.1 (3)	C6—C7—C2—C3	1.8 (4)
C8—N2—C7—C2	178.0 (2)	N2—C7—C2—C3	−179.5 (2)
C9—C8—C13—C12	−1.3 (4)	N2—C7—C6—C5	−178.7 (3)
N2—C8—C13—C12	178.7 (2)	C2—C7—C6—C5	−0.5 (4)
C11—C12—C13—C8	0.8 (4)	C2—C3—C4—C5	0.2 (4)
C2—N1—C1—N2	−0.2 (3)	C7—C6—C5—C4	−0.9 (4)
Co1—N1—C1—N2	176.35 (16)	C3—C4—C5—C6	1.1 (4)
C7—N2—C1—N1	0.0 (3)		

Symmetry codes: (i) −*x*, *y*, −*z*+3/2; (ii) −*x*+1, *y*, −*z*+1/2.
